# Passive Wireless Pressure Sensing for Gastric Manometry

**DOI:** 10.3390/mi10120868

**Published:** 2019-12-10

**Authors:** Alexander Benken, Yogesh Gianchandani

**Affiliations:** Center for Wireless Integrated MicroSensing and Systems (WIMS^2^), ECE Division, EECS Department, University of Michigan, Ann Arbor, MI 48109, USA; yogesh@umich.edu

**Keywords:** physical sensors, manometry, motility, passive, wireless, pressure

## Abstract

We describe a wireless microsystem for gastrointestinal manometry that couples a microfabricated capacitive transducer to a dual-axis inductor, forming a resonant inductor-capacitor (LC) sensor within an ingestible 3D printed biocompatible capsule measuring ø 12 mm × 24 mm. An inductively coupled external telemetry unit wirelessly monitors the pressure dependent resonant frequency of the LC sensor, eliminating the need for integrated power sources within the ingested capsule. In vitro tests in saline show pressure response of −0.6 kHz/mmHg, interrogation distance up to 6 cm, and resolution up to 0.8 mmHg. In vivo functionality is validated with gastrointestinal pressure monitoring in a canine beagle over a 26-hour period.

## 1. Introduction

Many industries can benefit from miniaturized wireless pressure monitoring, but it is perhaps the biomedical field which may have the most compelling needs [1]. Intraocular, intra-arterial, intracranial, and gastrointestinal applications demand miniaturized pressure sensing telemetry systems in order to provide physicians with critical patient data. 

The gastrointestinal (GI) tract is dependent on regular contractions to ensure consistent digestion and passage of food. Abnormalities in these contractions and the resulting pressure waves can result in, or result from, a number of motility disorders associated with nausea, abdominal pain, and vomiting [2]; detecting these irregularities is important for proper diagnosis and treatment. One tool is scintigraphy, requiring ingestion of radiological material to monitor gastric transit time [3]; however, this approach cannot detect any physical phenomenon within the GI tract. In order to more closely investigate the intestinal tract, pressure monitoring (e.g., gastric manometry) is often utilized. Traditional endoscopic techniques can be used, but due to instrument rigidity and size it can be difficult to image the small intestine and may cause pain and discomfort in some patients [4]. 

Another diagnosis regime employs pill-shaped ingestible devices, typically containing sensors, readout and data transmission electronics, and batteries [5]. Commercialized endoscopy pills include the Pillcam^TM^ (Medtronic, Minneapolis, MN, USA), which images the intestinal tract but lacks physical sensors [6], and the SmartPill^TM^ (Medtronic, Minneapolis, MN, USA), which records pressure, offering >100 mmHg pressure range, 10 mmHg pressure resolution, and 1 Hz readout bandwidth [7]. Ongoing research is directed at developing gas sensing ingestible pills capable of sampling oxygen, hydrogen, and carbon dioxide concentration in the gastrointestinal tract, which have completed successful human trials [8]. While these active ingestible pills have demonstrated effective functionality, passive telemetry approaches are attractive for potentially smaller form factors, greater longevity, simplified architecture to reduce cost, and eliminating the ingestion of undesirable materials (e.g., radiological and battery chemicals). 

A widely employed passive telemetry method utilizes the phase response of an inductor-capacitor resonant circuit (LC sensor), in which the resonant peak is altered by capacitively sensed pressure [9]. Prior work includes a capacitive pressure transducer combined with an implantable inductive “stentenna” used to measure intra-arterial blood pressure; however, the readout range and resolution are limited to 1 mm and 50 mmHg, respectively [10]. A commercially available LC sensor, the CardioMEMS^TM^ HF System (Abbott Laboratories, Lake County, IL, USA) was utilized to measure pulmonary artery pressure to monitor for congestive heart failure; it provides 320 mmHg pressure range, 1 mmHg resolution, and 200 Hz readout bandwidth [11].

**Figure 1:**
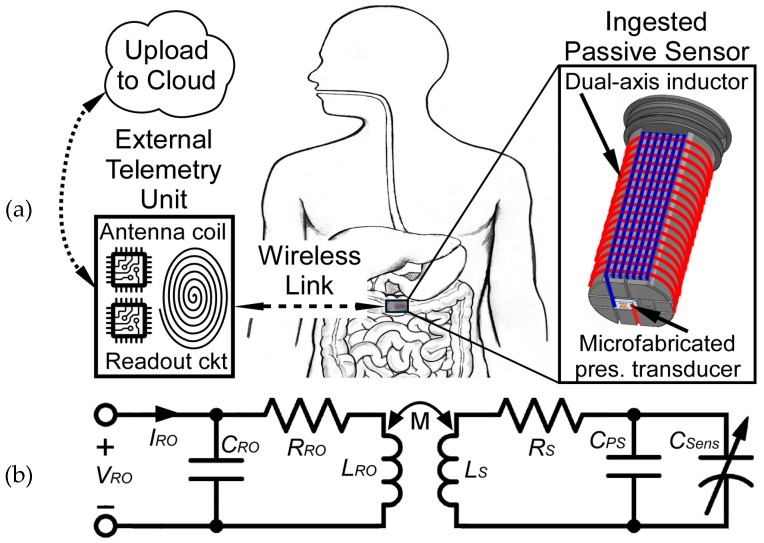
(**a**) Illustration of passive wireless pressure sensing system, showing ingested pressure sensor capsule, portable external telemetry for wirelessly monitoring and recording gastrointestinal pressure via inductive coupling, and proposed data upload to the cloud for remote review by attending physician. (**b**) Inductor-capacitor (LC) sensor circuit model illustrating input voltage and current, parasitic capacitances and resistances, and mutual coupling between LC sensor and external readout coils.

Our effort assesses an alternate method of diagnosing motility disorders that eliminates the need to ingest radiological or battery materials while simultaneously reducing sensor pill complexity, and potentially reducing the cost for this single-use disposable device. It realizes an ingestible capsule containing a passive LC sensor and inductively coupled external telemetry unit with control software for monitoring gastric pressure (Figure 1), focusing on maximizing interrogation distance, resolution, and readout bandwidth.

## 2. Methods and Modeling

### 2.1. Inductive Coupling Analysis 

A lumped model for the sensing system is shown in Figure 1b, where *C_RO_*, *R_RO_*, and *C_PS_*, *R_S_* are the parasitic capacitance and resistance of the readout coil and sensor, *C_Sens_* is the pressure transducer, and *M* is the mutual inductance between the inductors *L_RO_* and *L_S_*. Relevant design equations are summarized in Table 1. The complex input impedance, *Z_in_*, of the external readout coil is calculated from *V_RO_* and *I_RO_*, the complex input voltage and current (1). Hence, the frequency dependent phase of the input impedance, *ϕ_Zin_(f)*, is the difference between the phase components of *V_RO_* and *I_RO_* (2). At the resonant frequency of the LC sensor, *f_0_*, this phase is reduced relative to higher and lower frequencies. The size of this “phase-dip”, Δ*ϕ_PD_*, is indicative of the level of coupling, *k*, between the external coil and LC sensor, and is also affected by the quality factor, *Q*, of the LC sensor. The Δ*ϕ_PD_* at *f_0_* can be calculated using (3), where *Q* is calculated using (4) [12] and *k* is calculated using (5) [13]. The LC sensor resonant frequency, *f_0_*, is given by (6).

Although both *Q* and *k* affect Δ*ϕ_PD_*, *Q* also impacts the frequency span over which the phase-dip is evident. As the frequency span of the phase-dip increases, the minimum pressure resolvable by the system declines due to a degradation in the accuracy with which *f_0_* can be extracted through curve fitting of discrete data. The degradation of *Q* is primarily caused by series resistance, *R_S_*, of the LC sensor. The AC resistance of the coil, *R_S,Coil_*, can be estimated using equations that account for the skin-depth effect [14]. *R_S,Coil_* can be further increased as much as 5-fold due to the proximity effect with closely spaced (*S*) inductor windings [15]. The equivalent series resistance (ESR) of the capacitive transducer can be estimated using the sheet resistance and cross-sectional area of the thin film electrodes. 

Coil coupling, *k*, is affected by many parameters; if the readout coil is much larger than the sensor and well aligned to it, coupling is approximated by (5), where *µ_0_* is the permeability of free space, *n* and *r* are the turns and radius of each coil, and *z* is the coil separation (i.e., interrogation distance). Available equations [16] can be used to estimate coil inductances, *L_S_* and *L_RO_*. Using (3)–(6), coil design can be refined to maximize performance within the physical boundaries of the gastric manometry application. 

### 2.2. System Design and Fabrication 

#### 2.2.1. Sensor and Readout Antenna 

In this work, the planar external readout coil was constrained to a physical radius, *r_RO_*, of ≤7.5 cm (to permit ease of use during in vivo canine testing) and its self-resonant frequency, *f_0,RO_*, was constrained to be at least double of the LC sensor *f_0_* to prevent interference. Maximum coupling, *k*, at a given interrogation distance, *z*, can be achieved when *r_RO_* is equal to *z*. The maximum *r_RO_* of 7.5 cm was chosen to maximize the allowed value of *z*. To prevent *f_0,RO_* from decreasing below 25 MHz, a maximum *L_RO_* of 20 µH was set, assuming that the parasitic capacitance of the readout coil, *C_RO_*, was 2 pF.

**Figure 2:**
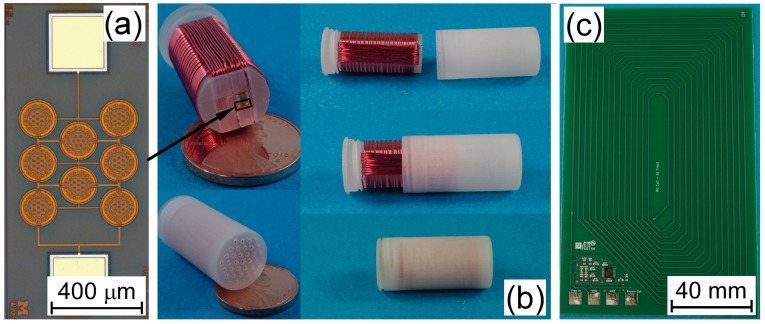
Optical images of (**a**) capacitive pressure transducer, (**b**) unpackaged and packaged LC sensor, and (**c**) readout coil and electronics.

Physical size constraints for the LC sensor footprint were set by the capsule interior volume at ø 10 mm × 20 mm. The sensor coil utilized a dual-axis design to improve coupling in non-ideal alignment scenarios, as sensor orientation cannot be determined once deployed. These z-axis and x-axis coils were connected in series to the pressure transducer, as shown in Figure 2b, to permit monitoring of a single resonant frequency, regardless of which axis is coupled to the readout coil.

The capacitive pressure transducer was fabricated on a dielectric substrate with features made from dielectric layers to enabled low offset and parasitic capacitances. This permitted an increase in Δ*C/C_0_* ratio, which enhanced LC sensor response (*∆f_0_/∆P*). It was designed to maximize capacitance change over 0–300 mmHg of applied gauge pressure (to fully cover pressure extremes within the gastrointestinal tract [17]) using the fabrication process described in Section 2.2.2. In this work, a non-ideal sheet resistance of the metal electrode resulted in a large ESR of ≈50 Ω. This has been mitigated with the placement of a low-ESR capacitor in parallel with the transducer. Although this negatively impacted the LC sensor response (*∆f_0_/∆P*), it reduced the ESR to <20 Ω, nearly doubling LC sensor *Q*.

The Δ*ϕ_PD_*, *Q*, and LC sensor response were calculated for all combinations of coil parameters within the boundary conditions. Due to the compromises associated with the LC sensor design, no one solution offers an optimal choice of maximum Δ*ϕ_PD_*, *Q*, and LC sensor response. For example, increasing LC sensor *Q* (by increasing the value of the low-ESR capacitor) will negatively impact LC sensor response (*∆f_0_/∆P*), as *∆f_0_* is inversely proportional to the offset capacitance, as shown in (6). A figure of merit was created by multiplying the Δ*ϕ_PD_*, *Q*, and *∆f_0_/∆P* to identify a design which balances all three parameters. The final design and measured device values are given in Table 2.

#### 2.2.2. Fabrication

The LC sensor support structure and housing were fabricated with 3D printing using a ProJet MultiJet printer and M3 Crystal, a biocompatible, acrylic-based resin [18]. The support structure consisted of an insert with guide grooves and a recess for the placement of the capacitive pressure transducer. The components were electrically connected with solder and then encapsulated in 2 µm of Parylene™. The LC sensor was secured in the protective housing and filled with SG-ONE Light Consistency Silicone Grease to ensure no air pockets were present. A grid of ø 500 µm holes were employed to permit pressure transmission to the pressure transducer. The final LC sensor is shown in Figure 2b.

A surface micromachining process was utilized to fabricate the capacitive pressure transducer [19]. It began with the deposition of a lower electrode (350 nm) on a dielectric substrate followed by plasma enhanced chemical vapor deposition (PECVD) of silicon nitride (100 nm) to provide insulation. Sacrificial amorphous silicon (α-Si) was used to define the inter-electrode cavity gap (*g* = 500 nm), and diaphragm diameter (ø 200 µm). The upper electrode (200 nm) was deposited by sputtering, followed by an initial diaphragm layer of PECVD silicon nitride and silicon dioxide (2.3 µm). Etchant access slits were opened and the α-Si was removed with XeF_2_. The diaphragm was sealed with PECVD dielectric (2.0 µm) and atomic layer deposition of Al_2_O_3_ (100 nm), *h* = 4.6 μm. Contact pads were opened with a dry etch and conventional dicing was used for singulation. Optical images are shown in Figure 2a.

## 3. Results and Discussions

### 3.1. Interrogation System 

In order to capture transient changes in GI pressure required for diagnosing motility disorders [17], the wireless interrogation system must measure the pressure-dependent resonant frequency, *f_0_*, with sufficient readout bandwidth (≥2 Hz) and calculate the corresponding pressure with adequate resolution (≤5 mmHg). The input impedance phase (*ϕZ_in_*) between the *V_RO_* and *I_RO_* was measured using a phase-to-voltage IC (Analog Devices AD8302) [20]. The required interrogation hardware was implemented with a National Instruments PXI-6115, which generated the input frequency voltage (*V_RO_*) and digitized the output of the AD8302. To meet the readout bandwidth and resolution requirements, a balance was calculated between the interrogated frequency range (IFR), the number of discrete interrogated frequencies (i.e., frequency step size, *f_ss_*), and the interrogation time at each frequency (see Appendix A for details). A LabVIEW^TM^ algorithm controlled the hardware using these parameters and extracted *f_0_* by applying a Gaussian fit to the phase-dip. 

Sample averaging was utilized to reduce noise and improve the minimum resolvable pressure. To permit a 2 Hz readout bandwidth at the necessary IFR and *f_ss_*, up to 12.5 ms is available at each frequency. System non-idealities such as frequency switching, data transfer, and curve fitting cause additional delays. Therefore, a total available averaging time of 5 ms is assumed, permitting up to 5 × 10^4^ samples at 10^7^ samples per second. When averaged, this can reduce uncorrelated white RMS noise by more than 200-fold [21]. 

### 3.2. Measurement Results 

**Figure 3:**
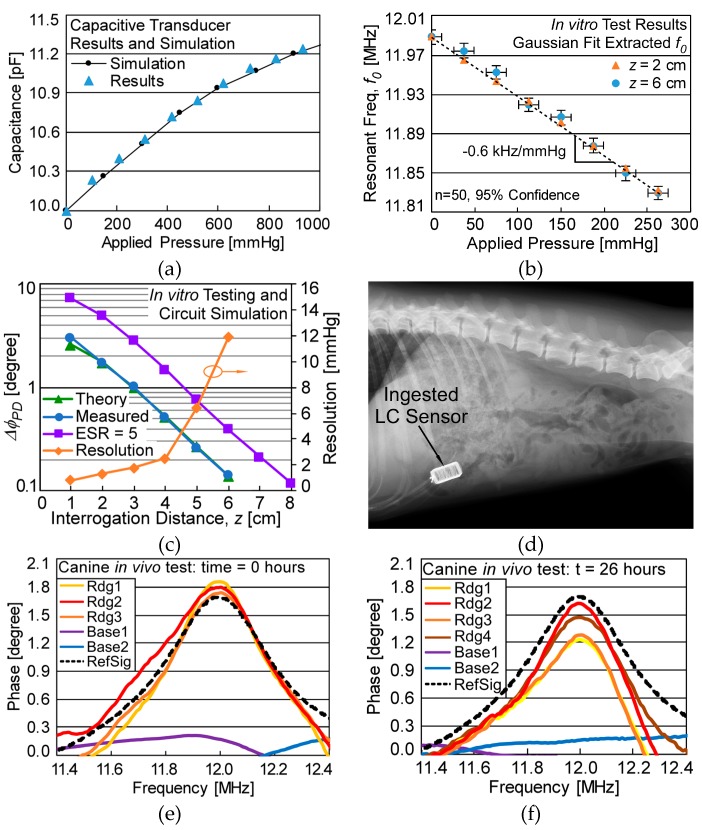
(**a**) Response of the capacitive transducer. Fitted simulation parameters: silicon nitride insulation: 75 nm, *g*: 0.45 µm; *h*: 4.5 µm; Young’s modulus: 80 GPa; RMS cavity roughness: 33 nm. (**b**) In vitro test results in a saline environment showing extracted resonant frequency, *f_0_*, as a function of applied pressure, error bars not visible for *z* = 2 cm. (**c**) Phase-dip size, Δ*ϕ_PD_*, vs. interrogation distance, *z*, showing measured, theoretical, and ideal (low-ESR) trends using the circuit of Figure 1b, and system resolution. (**d**) Radiograph of ingested LC sensor in canine specimen prior to in vivo interrogation. In vivo interrogation results (**e**) immediately after ingestion and (**f**) after 26 h residence in canine stomach, with reference overlay ‘RefSig’, the measured response in vitro at 0 mmHg gauge pressure and z = 2 cm.

The capacitive transducer and antenna inductor coils were independently characterized, then assembled in the LC sensor and tested in vitro in conductive saline to mimic deployment conditions. The same characterized device was then successfully deployed and interrogated in vivo in the GI tract of a canine model. The pressure transducer provided an offset capacitance of 10 pF and response of 1.8 fF/mmHg over an applied gauge pressure of 0–300 mmHg. Simulation parameters were fitted and found to be within the range of expected variation, given in Figure 3. An HP 4284 LCR meter was used to extract lumped values of the dual-axis inductor and readout antenna, listed in Table 2. 

Passive interrogation of the LC sensor used the system described in Section 3.1. These measurements showed a real-time readout bandwidth of 2 Hz while averaging 1024 sampled data points (which reduced uncorrelated white RMS noise only about 32-fold). Non-ideal frequency switching times in the PXI-6115 resulted in an irreducible delay of 10 ms per interrogated frequency, limiting the data collection period. For in vitro testing, the LC sensor was placed in an acrylic container filled with conductive saline (40,000 µS/cm) to mimic conductive losses that may be encountered during in vivo interrogation [22]. A syringe pump with an inline pressure gauge was used to pressurize the chamber. The measured *f_0_* as a function of applied pressure at *z* = 2 cm and *z* = 6 cm, shown in Figure 3b, demonstrates an LC sensor response of −0.6 kHz/mmHg. In vitro system resolution was assumed to be the 95% confidence interval of n = 50 extracted *f_0_* data points. The theoretical model and experimentally measured size of the phase-dip, Δ*ϕ_PD_*, and in vitro system resolution as a function of interrogation distance, *z*, are shown in Figure 3c. It should be noted that as Δ*ϕ_PD_* is a function of *z*, resolution could be determined through Δ*ϕ_PD_* when *z* is unknown, such as during in vivo interrogation. The predicted Δ*ϕ_PD_* with an almost ideal capacitive transducer (ESR = 5 Ω) is also given, presenting a maximum *z* of ≈8 cm for the same noise floor limit (Δ*ϕ_PD_* ≈ 0.15°). However, sensor *Q* would increase with a reduced ESR, permitting *f_0_* to be more accurately measured, further reducing the minimum detectable signal and thus increasing the maximum interrogation range. 

In vivo testing of the characterized LC sensor was performed on a beagle canine specimen (*Canis familiaris*) weighing ≈10 kilograms. Beagles are commonly used for bioequivalence studies of ingestible medication. The LC sensor was administered via mouth and radiographs taken prior to each measurement to verify LC sensor location, as seen in Figure 3d. The sensor was interrogated by holding the readout coil flush to the fur of the canine. Readings were taken by aligning the coil to the approximate known location of the sensor (labeled ‘Rdg#’ in Figure 3e,f) and at a location known to be misaligned with the sensor (‘Base#’). The reference measurement ‘RefSig’ in Figure 3e,f is the measured response in vitro at 0 mmHg gauge pressure and *z* = 2 cm. Sensor interrogation was completed immediately after ingestion (Figure 3e) and after a 26-hour residence period in the canine (Figure 3f); the sensor remained in the stomach for both readings. The *f_0_* was measured and, using the in vitro characterizations relating *f_0_* to applied pressure (Figure 3b) and *∆ϕ_PD_* to resolution (Figure 3c), the corresponding applied pressure and resolution were calculated for the in vivo readings, as shown in Table 3. 

After 26 h, the canine specimen ingested part of its bedding, causing it to regurgitate both the cloth and LC sensor, ending the in vivo experiment. The recovered LC sensor was re-tested and found to be functional, with no physical damage or change in *f_0_* or response. 

## 4. Conclusions

A passive wireless pressure sensing system was evaluated for gastric manometry in order to monitor GI tract pressure and aid in the diagnosis of motility disorders. The sensor was packaged into a biocompatible 3D printed ingestible capsule measuring ø 12 mm × 24 mm, utilizing inductive coupling telemetry to passively monitor pressure. A surface micromachined capacitive pressure transducer was coupled to an 11.0 µH dual-axis inductor coil forming the resonant LC sensor. It provided a response of −0.6 kHz/mmHg over applied pressures of 0–262 mmHg with a real-time readout bandwidth of 2 Hz, maximum interrogation distance of 6 cm, and pressure resolution of up to 0.8 mmHg in vitro. The sensor was successfully deployed and interrogated in vivo, demonstrating functionality in a canine GI tract over a 26-hour residence period. While in vivo testing was performed with a small planar coil to permit ease of use during in vivo canine testing, a larger coil belted around or draped on the torso is envisioned. Performance may be improved by reducing resistive losses of the capacitive transducer, which limited interrogation distance and resolution, as well as improving interrogation hardware to reduce switching delays in order to improve readout bandwidth and reduce noise. In general, a completely passive sensing system rivaling active GI tract sensors could be realized, increasing safety and reducing cost. 

## Figures and Tables

**Table 1:** Inductor-capacitor (LC) sensor design equations.

Parameter	Equation	
Complex input impedance	Zin=VROIRO	(1)
Input impedance phase	$\phi_{Zin}\left(f\right)=\phi_{VRO}\left(f\right)-\phi_{IRO}\left(f\right)$	(2)
Phase-dip size at *f_0_*, small *R_RO_*, *C_RO_*	$\Delta \phi_{PD}=\phi_{Zin}\left(f_{0}\right)-\phi_{Zin}\left(f_{0}/10\right)$ $\Delta \phi_{PD}\approx \arctan\left(Qk^{2}\right)$	(3)
Quality factor	Q≈(1RS)LS(CPS+CSens)	(4)
Coupling factor	$k\approx \left(\frac{\mu_{0}\pi }{2}\right)\left(\frac{n_{RO}n_{S}}{\sqrt{L_{RO}L_{S}}}\right)\frac{\;r_{RO}^{2}r_{S}^{2}}{{\left(r_{RO}^{2}+z^{2}\right)}^{1.5}}$	(5)
Resonant frequency	f0≈1(LS)(CPS+CSens)	(6)

*n_RO_*, *n_S_*: readout and sensor inductor coil turns; *r_RO_*, *r_S_*: readout and sensor inductor coil radius; *z*: inductor coil separation (i.e., interrogation distance).

**Table 2:** LC sensor and readout coil parameters and measured values. ESR: equivalent series resistance.

Parameter	Value
z-axis coil dimensions	ø 10 mm × 20 mm
z-axis: *n_S,z_*, *S_S,z_*	42 turns, 0.16 mm
z-axis: *L_S,z_*	6.3 µH (*R_S,z_* ≈ 4.5 Ω)
x-axis coil dimensions	9 mm × 19 mm × 5 mm
x-axis: *n_S,x_*, *S_S,x_*	15 turns, 0.05 mm
x-axis: *L_S,x_*	4.7 µH (*R_S,x_* ≈ 6.5 Ω)
Capacitive transducer, *C_Sens_*	*C_0_* = 10 pF, (ESR ≈ 50 Ω), Δ*C* = 1.8 fF/mmHg
Low-ESR Cap., *C_PS_*	*C_0_* = 5 pF (ESR ≈ 0 Ω)
Parasitic Cap., *C_par_*	≈1 pF
Inductor-capacitor (LC) sensor *R_S_*	30.5 Ω
LC sensor response	−0.6 kHz/mmHg
Readout coil size	9 cm × 15 cm
*n_RO_*, *S_RO_*	14 turns, 2.5 mm
Readout coil inductance, *L_RO_*	17.3 µH
System resolution, *z* = 1 cm	0.8 mmHg

**Table 3:** Measured *f_0_* and interpreted applied pressure during in vivo testing.

t = 0 h	Measured *f_0_*	Interpreted Pressure
Rdg1	11.9892 MHz	−0.8 ± 0.4 mmHg
Rdg2	11.9902 MHz	−2.6 ± 0.4 mmHg
Rdg3	11.9880 MHz	1.2 ± 0.4 mmHg
**t = 26 h**	**Measured *f_0_***	**Interpreted Pressure**
Rdg1	11.9879 MHz	1.4 ± 0.6 mmHg
Rdg2	11.9872 MHz	2.5 ± 0.5 mmHg
Rdg3	11.9870 MHz	2.9 ± 0.6 mmHg
Rdg4	11.9870 MHz	2.9 ± 0.5 mmHg

## Appendix A

The IFR must be wide enough to capture the full phase-dip. To determine the IFR, an approximate Gaussian function for the phase-dip was assumed. The *f_0_* and *Q* can be related to the mean, *µ* (i.e., *f_0_*), and standard deviation, *σ* (i.e., *f_0_/Q*), along with a magnitude multiplier, *A*, and substituted into a Gaussian function, as shown in (A1).

(A1) Phase-dip≈A exp(−(f−f0)22(f0Q)2)

For this Gaussian approximation to capture >95% of the phase-dip, an IFR of four standard deviations (*f_0_/Q*) is required. Additionally, the span of the resonant frequency shift due to applied pressure on the LC sensor must be included. The required IFR is given by (A2),

(A2) $$\mathrm{IFR}=4\left(\frac{f_{0}}{Q}\right)+\left(f_{0}-f_{0,min}\right)$$

where *f_0_* is the resonant frequency of the LC sensor at zero applied pressure and *f_0,min_* is *f_0_* at maximum applied pressure.

The *f_ss_* determines the number of data points for which the Gaussian will be fit. However, curve fitting performance saturates beyond a certain data density. Empirical experimentation with the LabVIEW^TM^ Gaussian curve fitting function revealed that performance saturation was attained at 10 discrete interrogated frequencies within the phase-dip frequency response bandwidth (*f_0_/Q*). Therefore, the required frequency step size is given by (A3).

(A3) $$f_{ss}=\frac{\sigma }{10}=\frac{f_{0}}{10Q}$$

The required IFR and *f_ss_* are ≈1.0 MHz and ≈25 kHz, respectively, when values for *f_0_* (≈12 MHz), *Q* (≈50), LC sensor response (0.6 kHz/mmHg), and maximum pressure (300 mmHg) are utilized.
